# Paresis and Cerebral Syphilis1Read before the Section on Medicine, Buffalo Academy of Medicine, November 13, 1900.

**Published:** 1901-04

**Authors:** Arthur W. Hurd

**Affiliations:** Buffalo, N. Y., Superintendent Buffalo State Hospital


### BUFFALO MEDICAL JOURNAL.

VOL. XL.—LVI. APRIL, 1901. NO. 9.

### Original Communications.

### PARESIS AND CEREBRAL SYPHILIS.[1]

BY ARTHUR W. HURD, A. M., M. D., Buffalo, N. Y.,
Superintendent Buffalo State Hospital.

PARESIS, or as it is often called, general paralysis of the insane or paralytic dementia, in its ordinary manifestations is well known and needs no review. The classical type, beginning with the alteration in character and change in disposition, extravagant delusions, ataxia of speech and gait, with convulsive seizures, possibly periods of intermission, but through it all a progressive mental and physical degeneration, involving both the brain and spinal cord, is too familiar to need any rehearsing in a short paper; but I shall first speak of the resemblances and differences between cerebral syphilis and paresis, a distinction which is important from the points of diagnosis, prognosis and treatment, which everyone must admit who has much to do with such forms of disease, and especially those who through carelessness or unfamiliarity with the disease have passed well marked subjects of paresis for life insurance, as has often happened, only to be chagrined by their patients breaking down soon with most marked manifestations of a fatal disease. The differences in typical cases of each are many, but in atypical cases the manifestations are so nearly alike that differential diagnosis, no matter how important it may be, is often difficult and frequently impossible.

In passing, however, I wish to note one fact, as it raises a question which I hope will be settled sometime. That syphilis is often a cause of paresis no one will deny. That it may cause other symptoms which do not correspond to the usually accepted manifestations of paresis we shall see, and that a common cause should produce forms of disease which resemble each other is not surprising. We know that the virus or toxin of syphilis produces immediate lesions.

1. Read before the Section on Medicine, Buffalo Academy of Medicine, November 13, 1900.

When upon the skin or mucous membrane it usually acts quickly after infection, within a few weeks or months, but when it produces paresis or when it affects the nervous system generally, the duration is extremely long. Many cases of paresis come to us with histories of syphilitic infection of five, ten, fifteen and even twenty years standing; years and years after all of the manifestations have subsided and when the patient thought himself absolutely recovered.

Why syphilis is so long in attacking the nervous system and so quick to attack the cutaneous system, we do not know, but Alexander McLean Hamilton has raised the query which I simply wish to mention in passing, "whether there are any varying toxins in syphilis, some more active, which attack the skin and mucous membrane, and the like, and show immediate effects, and another toxin which has as yet eluded us, which lies dormant for many years until it finally shows itself, in either disease of the brain or spinal cord." This we hope will be elucidated, for the fact of paresis with a history of specific infection of from five to twenty years standing is too common to be dismissed; there must be some causal relation between the two. Is there not, therefore, some toxin as yet unknown to us which differs from the ordinary poison of syphilis, which is of this extremely slow but very forceful growth?

After considering the differential diagnosis between cases of ordinary paresis and cerebral syphilis, as it is ordinarily seen, I wish to note one form of cerebral syphilis, which I am inclined to believe is not common, but which from its course is likely to involve one in a mistaken prognosis, and also to discuss the influence of trauma in incipient paresis. This relation of syphilis to paresis has been very extensively discussed and studied and has even formed the subject of a symposium at an International Congress.

It is readily agreed that syphilis may be the cause (1) of any of the simple psychoses, aside from paresis; (2) that it may cause true general paralysis or give rise to certain similar symptoms, a clinical picture which has been denominated pseudo-paresis.

First.—Like any form of illness it may cause mania, melancholia or dementia.

Second.—It is generally acknowledged that syphilis may be the cause of true paresis and in a large proportion of cases apparently is. The third condition—namely, pseudo-paresis is what we wish to consider first. That the two diseases may approach each other and sometimes become confused is quite possible, especially in those cases in which the clinical picture in paresis is somewhat toned down, and in others in which the symptoms of cerebral syphilis are somewhat heightened. There are some which so closely approach each other as to lead some observers to deny practically any difference, but others vary so much as to be quite marked.

There is a difference in the mode of onset, and in specific diseases marked motor disorders usually precede the mental symptoms, while in paresis the reverse is usually the case. In specific disease also a temporary hypochondriacal or acute delirium may usher in the disease, while in paresis there is much more prolonged stage of onset, involving change in disposition, tastes, habits, and the like. Early paralyses of some of the ocular muscles, with difficulties of eyesight, optic neuritis, headache, nocturnal, deep, very persistent and urgent, local anesthesia, vaguely distributed, and neuralgia are much more common symptoms in specific cases. There are also differences in the general appearance and in the apparent condition of nutrition in the two cases. The paretic is usually of a comparatively good color, while the syphilitic cases show marked cachexia and anemia, even in the early stages. There is an irregularity in the appearance of the symptoms which is not found in paretic cases.

In paresis there is a fibrillary twitching of the tongue or facial muscles and hesitancy of speech quite characteristic, but if these are not fully developed we will derive but little help from them. In specific cases, however, the tremors and twitchings are more of a paralytic nature and have less of the ataxic in their character, and show less incoördination. The same is true of the existence of local muscular contractions and rigidities, and paralyses following convulsions are apt to be much more persistent in cases of specific disease; whereas, after convulsions the paralyses in paresis are frequently of a transient character.

The motor impairment in paresis is much more general and less localised and has an irregular course of progress, as distinguished from a more localised unilateral and constant paralysis in specific disease. Delusions of grandeur are much less pronounced, as a rule, in syphilis than in the paretic. One of the most diagnostic features, however, is, I think, the appearance and expression of well-being and of complacent groundless satisfaction with himself and his surroundings, which is at some time or other usually manifested in the paretic. It is something different from and more than the vacant expression which is often seen in specific dementia.

I cannot dwell on this phase of the subject at greater length, but will quote Mickle's table of points of differential diagnosis, acknowledging the great indebtedness of the profession to him for his work on this subject.

(*a*) The distinct history or symptoms of syphilis.

(*b*) The preceding cranial pains, nocturnal and intense.

(*c*) The exaltation, less marked, less persistent, and perhaps less associated with general maniacal restlessness and excitement than in most of the cases of general paralysis.

(*d*) Sometimes such complications as palsies of one or several cranial nerves, or hemiplegia, or paraplegia, having the character and course of syphilitic palsies.

(*e*) The greater frequency of optic neuritis, early amaurosis, deafness, local anesthesia, vertigo or local rigid contraction.

(*f*) The affection of articulation, paralytic rather than paretic, and usually the speech, which is not accompanied by any facial or labial tremors or twitchings.

(*g*) A frank cerebral or spinal meningitis or pachymeningitis.

(*h*) The variety of the motor and sensory symptoms.

(*i*) The effect of antisyphilitic treatment.

One of the strong points of differentiation remains to the last, and that is the variation in the course of syphilis, the prolongation of the disease beyond the average true paresis, the different results of specific treatment in cases with an avowed specific history and with lesions apart from the cerebral.

How syphilitic poison may be the cause of paresis years after infection and what the exact pathological progress has been in the brain, has been one of the most intricate and difficult of studies. While we know that the gross syphilitic lesions of the brain not paretic have a tendancy to affect the basal ganglia or cranial nerves, the bloodvessels and the meninges, both cortical and basal, yet in what way the changes in the brain in paresis of supposed syphilitic origin take place, has been a cloudy subject. One of the most important contributions to this question, however, has been given by Dr. Mott, pathologist of the London County Asylums, and has won attention from neurologists. I will endeavor to give a brief abstract of one of his discussions on the subject.

Mott's idea of the pathology of these parasyphilitic affections is that syphilis, whether acquired or inherited, may lower the specific vital energy of the nerve cells, so that the system of neurons may, under the influence of stress, in one form or another, die prematurely. He agrees with others that general paralysis is primarily a degeneration of the neuron with secondary changes in the membrane and vessels, in consequence of irritation from the products of degeneration. There are certain facts which support this view pathologically; certain regions of the brain are affected more than others; for instance take the central and frontal convolutions which generally show marked pia-arachnoid thickening, in comparison with the temporal and occipital lobes.

This has been associated by Flechsig with the distribution of the internal carotid artery. He considers that this pia-arachnoid thickening more closely corresponds with the distribution of veins which open into the longitudinal sinus, because in these veins the current is opposed to gravity, and on account of their oblique opening into the sinus and contrarily to the current in it, the area which is drained by them would be more likely to suffer from venous stasis, than the areas drained by the lateral sinuses and the torcula. Furthermore, he has found when nervous tissue breaks up, various products are formed which escape into the cerebro-spinal fluid, cholin, among others, and nucleo-proteid.

The existence, however, of various abnormal products in the cerebro-spinal fluid may explain the proliferation of the neuroglia elements; the nucleo-proteid constituent would suffice to explain venous stasis in areas undergoing degeneration, for it is known that this substance when injected into the circulation, even in small quantities, causes coagulation.

The dictum of Virchow "that a cell nourishes itself and is not nourished," is to Dr. Mott the fundamental principle of the pathology of general paraylsis. The complex nerve cell, termed the neuron, may undergo degeneration for two reasons: first, because the nutriment brought to it is inadequate, as, for example, in those forms of organic brain disease which arise from occlusion of arteries, there is obvious cause why the nervous units should cease to perform their functions; second, there is a reason why, when this cause is removed, the nerve cells will be restored in great measure to their original functions. But if the life of the nerve cell is coming to a close, it is of no use to bring fluid to nourish it, for it cannot assimilate it; therefore it dies prematurely. These degenerations are usually systemic; in the case of tabes the first afferent system of neurons is affected; in general paralysis it is usually the highest and latest developed association system of neurons which suffers first, but occasionally in the tabetic form it may begin in the first afferent system.

There is one striking feature in the pathology of general paralysis which is constant and that is that those delicate fibers which serve to coördinate afferent and efferent tracts, and association systems of the brain—that is the tangential system of fibers—are always wasted first. The syphilitic toxin is one of many factors in the production of the degenerative process, although not absolutely essential; at any rate, the statement "no syphilis no general paralysis" is not proved, but everything tends to prove that syphilis is the most important factor by reason of its prevalence, its persistence, and its potency in producing devitalising effects upon structures which are incapable of repair, and incapable of regeneration. The nerve-cells are perpetual elements; they exist when the body is developed at birth; there is no increase in number, there is merely an increase in complexity of structure. The neuron consists of a little mass of protoplasm which sends out one process, the neuraxon, and numbers of other processes, termed dendrons, until we have a structure very like a tree.

The syphilitic poison by its devitalising effects slowly produces a regressive metamorphosis, commencing at the terminal twigs and proceeding back to the trophic center, the cell body. If this be a slow process, involving only a small portion of the nervous system, as in tabes, autointoxication is less likely to occur from the breaking down of nervous matter. In rapid cases of general paralysis, where a large mass of nervous matter may be disintegrating, a vicious circle is liable to be established by the accumulation of products of degeneration in the perivascular lymphatics, causing vascular stasis and inflammation, which may be the exciting cause of fits and acute destruction of nervous matter. This seems highly probable, seeing that in cases where there have been prolonged epileptiform convulsions evidences of extensive degeneration in the crossed pyramidal fibers, upon examination of the spinal cord by the Marchi method, have been invariably found, proving therefore recent death of the psychomotor neurons of the Rolandic area. The fits were the evidence of the irritative discharge of the dying structures. This is the substance of Dr. Mott's discussion of the subject.

It will be noticed that Dr. Mott agrees with many other observers, that the specific poison is not the only factor, but to this must be added stress, and that is I think the common observation of all of us—that plus infection there usually is stress, worry, strain, etc., for in certain countries, for instance, in Mohammedan countries, where syphilis is quite prevalent, but where there is little push, anxiety or mental strain, paresis is extremely rare.

We know that syphilis of the brain manifests itself, generally speaking, in three different locations:

1. Along the basal ganglia, involving some of the nerves of special sense and some that preside over the motor apparatus of the eye.

2. Involving the bloodvessels.

3. The meninges.

There is a clinical manifestation of the disease which is quite rare, I am inclined to believe, and which may present itself in its fulminating character, de novo, or may arise in the course of one of these other syphilitic pathological situations; this is a condition in which the patient, after a more or less prolonged prodromal period, becomes suddenly and acutely worse with the symptoms of cerebral inflammation of a low grade. Without apparent pain or headache or photophobia, there are yet some of the symptoms of meningitis, but lacking some of the marked characteristics thereof. There may be extensive delusions, but they are fleeting and changeable and partake more of the nature of delirium. There is flushed face, great restlessness and excitement, and a temperature but moderately elevated, ranging in the neighborhood of 102°. This condition in its very active manifestations might be taken for a simple meningitis were it not for the known specific history of the patient. There are no paralyses of any muscles nor apparent disturbance of sensation.

With some of the symptoms of meningitis lacking the condition, is a puzzling one, but it seems possible and even probable that this is a true localised inflammation of the brain substance, or at least a meningo-encephalitis, distinct from the motor tracts and the basal ganglia. The condition is a rare one. It is mentioned by Dercum,[*] who remarks its rarity, says that it may develop during the course of an infectious disease as well as during syphilis, and he emphasises the lack of pronounced symptoms. Of course I do not refer to such pronounced cases as those of traumatic origin, which eventuate in abscess of the brain.

The cases which I have had opportunity of seeing have been few, but have been characterised by quite a distinct clinical picture. In one an autopsy was not obtainable. The patient after a few months of irritability, excitement, nervousness, sleeplessness, emaciation and extravagance characteristic of paresis, suddenly developed a low febrile condition, a muttering incoherence and exhaustion, dry tongue, but no paraylses, ocular or otherwise, no apparent pain or photophobia. This condition continued with marked emaciation for a few weeks when death ensued. Prognosis in this case was an important one, both to the family and to business associates and had been guarded from the first.

Another case, in which cerebral syphilis had been diagnosticated for a year and the patient had been under treatment at his home, developed this same condition of low febrile delirium without paralyses, pain and the like, as noted before. With active specific medication, however, the patient recovered sufficiently to return to his home. He still has periods of epileptiform unconsciousness and will undoubtedly go on to a fatal termination, but the local condition seems to have been relieved for the time being.

There seems to be no reason, except for the tendency of a toxic substance to attack spaces directly communicating with the lymphatics and the arterial system, why this specific toxin should not attack the brain substance itself. That it does not do so more often is probably due to the anatomical arrangement of the vascular supply, but that it is possible to do this and to be very sudden and fulminating in its character is a contingency which may always be looked for. That this is a sudden termination of paresis of syphilitic origin is a constant possibility.

Another instance of sudden termination in paresis, in what seemed to be an ordinary case, has come to my notice recently and was of importance from the standpoint of an insurance company, as the duration for a case of paresis was unusually short. In this case the patient, who had the muscular and mental symptoms of paresis for a few months, showed great emaciation and finally died very suddenly. It is not known that the patient had a specific history, although it is quite possible. The shortness of the duration, naturally, called in question the diagnosis, but at an autopsy a thrombus of the basilar artery was found, which was undoubtedly the immediate determining cause of death. That thrombi occurring in smaller arteries should produce temporary paralysis is a common experience, but that in a case of paresis of specific origin the thrombus may fill so important an artery as the basilar and cause sudden death early in the history, is a point well worth remembering.

Another point in paresis which I spoke of first and which has been referred to in a recent article by Dr. Wm. C. Krauss, of this city,[1] and which has come to my observation quite forcibly, is the effect of an injury occurring to a person suffering from incipient or even unrecognised paresis or cerebral syphilis in developing the disease. A careful inquiry has developed the fact that some patients who had symptoms of paresis very slightly had, immediately after

1. BUFFALO MEDICAL JOURNAL, October, 1899.

meeting with some accident, developed it; in fact, it seemed to cause the disease to blossom out.

In one case which came under my observation the patient was knocked down in the street and in a few days had every symptom of paresis, and this went on to a fully developed paresis, running the regular course, followed by death. Careful investigation led to the fact that he had shown some mental alteration before the accident. Further investigation led to the conclusion that he had been affected by syphilis years before and that the accident was simply the exciting cause of rapid development; a ripening influence which brought forward a result which would have been inevitable under any circumstances, but possibly postponed except for the trauma.

J. R., night watchman, in May, while in the pursuit of his duty was shot, being at the time in supposedly perfect health. Immediately after the shooting was confined to bed and during this confinement began to show mental disturbance, irritability, nervousness, sleeplessness, wandering, extravagant delusions, and the like. On being able to go about, he wandered about the streets looking for imaginary wealth, unable to concentrate his attention on anything for any length of time or to work. He was admitted to the hospital in September of the same year in an advanced stage of paresis and died in December of the same year, within eight months of the time when he was shot. This length of duration, of course, makes it improbable that his paresis started after the shot, but undoubtedly the accident simply served to bring it to the surface and to develop it.

Thus it is seen that without a careful study of the disease the accident or injury might be considered the cause of the paresis and damages sued for accordingly; whereas, in fact, the paresis was an underlying condition which the accident simply served to bring rapidly to the surface, and this is also true as a common experience in cases of syphilis.

Another interesting fact, and one which bears out the line of thought which I have endeavored to present here, is the influence which an attack of ordinary insanity may have in paving the way for a true paresis. Many patients after a typical attack of mania or melancholia, with a recovery or apparent recovery, come back after a few years of what is alleged by friends to be perfect health, with the symptoms of paresis well marked. It seems in perfect accord with the pathology of the subject which we have just studied, that the devitalising and depressing influence of the first attack on the brain has paved the way for a genuine paresis.

I might refer briefly to one test which has been suggested by Justus for the recognition of syphilis, in 1895. He asserted that a single inunction of mercury in all untreated cases of syphilis beyond the first stage, causes a reduction in the hemoglobin due to the sensitiveness of the red blood corpuscles to the drug, while in those not infected no reaction follows. Percentage of hemoglobin is determined first, then 40 to 60 grains of unguentum hydrargyri is used as inunction, and after an interval of some hours—24 to 72—the hemoglobin is then examined. There are some conditions which render the test not infallible.

It has been said that chlorosis may show some reaction, and it might be that other nonsyphilitic conditions may also. It is also said to fail in two classes—namely, in latent and early cases. The range of usefulness does not seem to be entirely worked out. In two of our cases I would say that the test was negative, but the course of the disease indicated that the patients did not have syphilis, and the negative, in so far as a negative test is of value, was in keeping with the subsequent clinical history. It is probable that this test, like many others, has fields of usefulness as an aid and assistance, but is not universally applicable or infallible.

With all our aids to diagnosis there still remains a certain number of cases in which the diagnosis must be in doubt and must be assisted by the effects of anti-specific treatment, and this has its limitations, for the test must be made before the brain syphilis, if it is brain syphilis, has produced marked pathological changes in the lumen of the vessels, deposits of inflammatory material and the like, which although the results of syphilitic virus, are not specific in themselves and are not affected by treatment.

